# “*But at a certain point, the lights literally went out*”: A qualitative study exploring midlife women’s experiences of health, wellbeing, and functioning in relation to paid work

**DOI:** 10.3233/WOR-220567

**Published:** 2024-03-08

**Authors:** Marjolein Verburgh, Petra Verdonk, Maaike Muntinga, Irene van Valkengoed, Carel Hulshof, Karen Nieuwenhuijsen

**Affiliations:** a Department of Public and Occupational Health, Coronel Institute of Occupational Health, Amsterdam Public Health Research Institute, Amsterdam UMC, University of Amsterdam, Amsterdam, The Netherlands; b Department of Ethics, Law and Humanities, Amsterdam Public Health Research Institute, Amsterdam UMC, Vrije University, Amsterdam, The Netherlands

**Keywords:** Women, work, middle aged, menopause, mental health, qualitative research

## Abstract

**BACKGROUND:**

In the Netherlands, the fact that midlife women constitute a considerable segment of the working population is relatively new. Generally paid work contributes to midlife women’s wellbeing, but they also report health challenges, such as work-related fatigue and the menopause.

**OBJECTIVE:**

The objective of this study is to understand how midlife women themselves perceive their health, wellbeing, and functioning in relation to paid work.

**METHODS:**

In this exploratory qualitative study, 28 women participated in five ethnically homogeneous focus group discussions (FGDs). De FGDs were recorded, transcribed verbatim, and thematically analyzed using MAXQDA.

**RESULTS:**

We identified exhaustion as central to our analysis. During midlife, exhaustion seems to occur once a certain limit has been reached, both physically and mentally, with women feeling to have reached the end of their rope. Besides obvious physiological challenges, we identified two major themes in which we discuss challenges both in paid work and private life: (1) *work environment and working conditions*, and (2) *burdens in private life*. Participants took various measures to manage and try to reduce exhaustion, including finding a new job or negotiating different job tasks, and reducing work hours.

**CONCLUSION:**

This study indicates that the extent to which women experience exhaustion is associated with challenges in both paid work and private life. The underlying processes do not seem to reflect individual problems, but reflect a complex set of factors at the structural level. Nevertheless, women take several individual measures to reduce their exhaustion, including reducing their participation in paid work.

## 1 Introduction

In the Netherlands until the mid-20th century, women were forced to quit their paid job when they married and had children and as a consequence, older women were scarce in the labor market [1]. Increasing numbers of women are active in the labor market during midlife; more than 1.8 million Dutch women aged 45 or older are currently in paid work [2]. Older workers, women included, have more chronic health problems and report more often work-related health issues than their younger counterparts [3], and with menopause as a female-specific life phase we were interested in how midlife women themselves perceive their health, wellbeing, and functioning in relation to paid work.

Although paid work generally contributes to the wellbeing of midlife women, for example by providing women with a meaningful identity [4], several work-related threats to their wellbeing can also be identified. Work-related fatigue is more common among highly-educated midlife women than among other groups [5]. Compared to men, women are more often exposed to risk factors for work-related fatigue, such as high work pressure, low work autonomy and high emotional work demands [5, 6]. This can be explained by the fact that women work relatively more in female-specific sectors, such as healthcare. In 2021, the Netherlands health care system had the highest incidence of reported occupational diseases, with more reports of occupational diseases in women (62%) than in men (38%) [7]. Psychological occupational diseases were the second largest group [7], which often lead to long-term sickness absence [8].

In addition to work-related threats comes the menopause^1^. Growing numbers of studies provide evidence that menopause may negatively impact work-related outcomes (e.g., work ability [9–17], productivity [15, 16, 18], and sickness absence [15, 16, 19–22]. Geukes et al. (2012, 2016), show that in the Netherlands menopausal symptoms are associated with a lower work ability [11, 17], and that women with severe symptoms in particular experience serious work ability problems [17]. Unfortunately, most international studies on menopause and work almost exclusively look at this phenomenon from a biomedical perspective. Work ability and similar work-related outcomes must be understood in the context of women’s lives. The menopause in itself may not be a problem, but the context in which it takes place can make it feel like a major challenge, consequently affecting work-related outcomes. It has been shown that the experience of menopause in the workplace can be accompanied by stereotyping, taboo, shame and fear [23–25].

Besides the menopause, women experience many other sex- and gender-specific challenges over the course of life which impact their wellbeing. Following Dillaway (2020) who states that we probably blame menopause for more than we should, we argue that physiological challenges, must also be related to the context of women’s lives, their specific life situations, their lifestyle behaviors, and to chronic conditions they may suffer from [26]. For instance, Dutch statistics on informal care show that midlife women carry the burden of long-term informal care work almost twice as often than men of the same age. This adds to their more adverse working conditions and to the burden of menopause [27]. Long-term informal care for people with chronic disease or impairment is associated with increased risk for psychological fatigue [28].

So far, studies on menopause and work have been are predominantly quantitative, and the perspectives of midlife female workers have hardly been examined qualitatively [23, 24, 29]. Contextualizing the experiences of women from their own perspective can help work organizations to provide the right tools to support midlife women and to enable more inclusive work environments for female workers at this stage of life. Therefore, in this qualitative study, we addressed the following research question: How do women in midlife perceive their health, wellbeing, and functioning in relation to paid work?

## 2 Materials and methods

### 2.1 Study design

This study followed an explorative qualitative study design, with content analysis as methodological orientation. We used focus group discussions (FGDs), as an appropriate method to explore a wide range of perceptions, feelings, and experiences [30]. Since in FGDs the emphasis is on the interaction between participants [31], this was also an appropriate method to explore how women reacted to each other; whether there were shared experiences and whether their experiences differed.

### 2.2 Reporting guidelines

We used the consolidated criteria for reporting qualitative research (COREQ) to comprehensively report the focus group process [32].

### 2.3 Participants

Women were eligible to participate if they were aged between 45 and 60 and did paid work for at least 12 hours a week or had worked until at least the age of 45. To explore a wide range of experiences, we aimed to include women from different ethnic backgrounds (majority and largest minorities in Amsterdam): Dutch, Moroccan, Turkish, Surinamese, and Ghanaian. Recruitment was through social media, collaboration with a migration women’s organization, and snowballing through informal contacts. Participants received a 25 euro gift card in return. Since we had quite a lot of interest from native Dutch women, we purposive-sampled across age, profession, contract hours, educational level, and living situation in this subgroup. Due to the recruitment challenges associated with COVID-19 measures, we convenience-sampled the other groups. Interested women received information by detailing the background and purpose of this study. Due to language barriers, most Ghanaian participants were given information at a face-to-face meeting.

We included 28 participants, and organized five ethnically homogeneous FGDs, because women tend to open up more in the company of women with the same ethnic background who might have similar cultural experiences. To prevent bias, we paid attention to differences within the FGDs. We mainly organized constructed groups where participants had not previously met. Only the Ghanaian and Moroccan groups were natural groups where most participants knew each other beforehand. All women who agreed to take part in the study actually participated. Table 1 gives descriptions of the participants.

**Table 1:** Description of respondents

N	*Ethnicity^1^*	*Age*	*Profession*	*Contract hours per week*	*Educational level^2^*	*Living situation^3^*
1	Dutch	52	Costumer care support employee	20 hours	Intermediate	Partner and two children
2	Dutch	48	Owner small childcare at home	30 hours	Intermediate	Partner and one child
3	Dutch	46	Administrative assistant	28 hours	Low	Partner
4	Dutch	58	Project secretary	36 hours	Intermediate	Two children
5	Dutch	54	Financial employee	32 hours	High	Partner and two children
6	Dutch	56	Teacher	32 hours	High	Partner
7	Dutch	50	Teacher	32 hours	High	Two children
8	Moroccan	54	Project leader small government organization	24 hours	Intermediate	Partner and two children
9	Moroccan	50	Director small government organization	36 hours	High	Partner and one child
10	Moroccan	51	Primary school bus driver	15 hours	Low	Partner and one child
11	Moroccan	49	Cook small government organization	36 hours	Intermediate	Two children
12	Antillean	52	Stylist	38 hours	Intermediate	One child
13	Surinamese	57	Ambulatory care worker	32 hours	High	Single
14	Surinamese	55	Financial administrator	36 hours	High	Single
15	Surinamese	59	No paid job (owner foundation)	5 hours	Intermediate	Single
16	Surinamese	49	Manager assistant	36 hours	Intermediate	Two children
17	Surinamese	49	Patient food service assistant	30 hours	Intermediate	Two children
18	Surinamese	56	HR employee- coordinator volunteers	36 hours	High	One child
19	Ghanaian	50	Cleaner	35 hours	Low	Partner and two children
20	Ghanaian	43	Cleaner	Unknown	Low^4^	Single
21	Ghanaian	53	Cleaner	32 hours	Low	Partner and three children
22	Ghanaian	48	Cleaner	25 hours	Low	Partner and three children
23	Ghanaian	54	Cleaner	32,5 hours	Low^4^	Alone
24	Turkish	47	Associate professor	36 hours	High	Partner and two children
25	Turkish	43	Social worker	28 hours	High	Partner and two children
26	Turkish	54	Home care worker	16 hours	Intermediate	Partner
27	Turkish	48	Management assistant	26 hours	Intermediate	Partner and two children
28	Turkish	52	Department secretary	38 hours	Intermediate	Single

^1^Based on the Dutch Central Bureau of Statistics (CBS) criteria, we defined ethnicity according to the country of the birth of the participant as well as her parents. A participant was considered non-Dutch if she was born abroad or having at least one parent born abroad, or being born in the Netherlands but both parents being born in abroad. ^2^Based on the Dutch CBS criteria, we defined low educational level by completion of primary school, lower vocational education, and lower secondary school, intermediate educational level by completion of intermediate vocational education and upper secondary school, higher educational level by completion of upper vocational education and university. ^3^Living situation really only concerns which persons live at home with the respondent. Children who no longer live at home, or a partner living separately are therefore not included here. ^4^The educational level is an estimation as these respondents did not share their educational level during the focus group discussion.

### 2.4 Ethical considerations and informed consent

All methods carried out in this study involving human participants were performed in accordance with relevant guidelines and regulations (1964 Declaration of Helsinki). The research proposal was submitted to and approved by the Medical Ethical Committee of Amsterdam UMC, location AMC, who decreed that a comprehensive evaluation was not required since this study was not subject to the Medical Research Involving Human Subjects Act (W20_381 # 20.426). Informed written consent was required from all individual participants included in the study. Prior to participation, each participant was adequately informed of the aims and methods of the study and written informed consent for participation in this study was obtained from all participants. No personal information has been used and the individual’s identity has been protected by removing any combinations of personal identifiers from the data. Pseudonyms were designated to the participants to guarantee their anonymity.

### 2.5 Procedures and data collection

We conducted five FGDs between November and December 2020. Due to COVID-19 measures, we organized online FGDs except for the Ghanaian and Moroccan groups which were face-to-face. We organized the FGD for Moroccan participants at a community center of an organization for migrant women in Amsterdam, and for the Ghanaian participants at an external and secluded location at the Amsterdam UMC. The moderator was the first author MV who is a PhD researcher. The FGDs were audio-recorded and field notes were taken by the last author KN. Both are white female interviewers and experienced qualitative researchers. Both authors are employed at the Amsterdam UMC. The authors did not know the participants before the FGDs, with the exception of four participants whom MV knows from a previous study. Apart from the researchers and participants, no one else was present.

Prior to the FGD, MV wrote a case story based on participant’s experiences collected in conversations by telephone or face-to-face. Participants were asked to name three things that are challenging or beneficial to continuing working during midlife. At the start of each FDG, MV explained that this study was part of a larger PhD project on women’s midlife and work. KN started the FGDs by reciting the case story. The primary questions posed in the FGD were: Who recognizes herself in this case story? Who wants to respond to this case story? Furthermore, we used a topic list (e.g., private situation, working conditions, life-stage-specific health) which was not pilot-tested. This is not an in-depth study on how women’s cultural backgrounds play a role, but rather a study to collect and explore a rich set of data on midlife women’s experiences with wellbeing in relation to paid work and private situation data across the largest ethnic groups in the Netherlands. Since we wanted to collect rich data, we were not after data saturation. All FGDs were held in Dutch, except for the Ghanaian group which was held in English. The average duration of each FGD was approximately 1.5 hours. Recordings were transcribed verbatim. We did not send the transcripts back to the participants for comments.

### 2.6 Data analysis

Data analysis was managed using MaxQDA. We used thematic analysis to identify relevant topics in three steps: (1) open coding, (2) axial coding, and (3) selective coding [33, 34]. First, each transcript was open coded by MV, and simultaneously one of the other authors independently coded fragments from one transcript. In total five authors coded transcripts. Disagreements about open coding were resolved by discussion between two authors. Second, MV built a coding framework comprising topics and subtopics and she re-conceptualized and clustered the codes. Third, after discussing this framework with all authors, central themes were identified. These themes were shared in a member check with the participants by e-mail, and for the Ghanaian participants by voice memos [35]. The majority reported that they recognized their experiences in the findings but did not add comments. Central themes were adjusted and refined based on these member checks and are described in detail below.

## 3 Results

In our analysis, we identified exhaustion as the central theme. During midlife, participants across all ethnic backgrounds experienced challenges, and the majority experienced exhaustion, which manifested itself in feeling tired or lacking energy, concentration problems, and irritability. Exhaustion seemed to only arise after a limit had been reached, i.e. both physically and mentally being ‘at the end of their rope’, as illustrated by the following quotes in which participants used varying words and phrases to describe their exhaustion:

“*Due to everything that has happened in my private life, and to the menopause **it all got too much for me** I couldn*’*t do it anymore, because here [at work] I did my best to be normal, but when I got home I felt completely drained.*” *(Nadia, 54, Moroccan, project leader small municipal organization for migrant women, 24 hours a week, 17112020)*

“*What triggered it in me is my husband had a serious accident three years ago. I worked a lot then **but at a certain point, the lights literally went out**. And it was indeed then that I was shown to be in the menopause.*” *(Hanneke, 46, native Dutch, owner small child care, 30 hours a week, 13112020)*

“*I recognize it [* . . . *] sleeping badly and caring for someone else, I mean, informal care. Irritable and burned out too. **That feeling that you are burned out,** I recognize it in myself.*” *(Helen, 57, Surinamese, ambulatory social worker, 32 hours a week, 25112021)*

These quotes are similar in that the combination of menopause with challenges in their private life appears to make them feel exhausted. Moreover, all these participants work in women-specific professions with high emotional work demands; a context that overwhelms them, and is not adapted to their specific needs. It was only in the Ghanaian group that participants did not speak of exhaustion in these kinds of terms. Marie (56) is a native Dutch woman who works as a teacher for 32 hours a week who suffered from several “*complaints*”, such as sleeping problems, fatigue and lack of energy, experienced this period in which she felt exhausted as a “major drama”, also because she was not aware of being menopausal. She reflected on the last decade of her life:

“*I had a burnout and I didn*’*t know I was in the menopause. I suspected it and said so a few times to my GP and to my therapist at that time. They said* ‘*Girl, you are only 46. You aren*’*t in the menopause yet.*’ [. . . *]. It took me a lot of time and energy to get through to them, and by the time I saw a menopause consultant I was 50 and it had been going on for about five years and she said I was right after all, I was in the menopause. I was given something for it and I thought* ‘*OK. I*’*m not crazy then.*’ *It was such a struggle at work to get the situation clear. It wasn*’*t like* ‘*oh, I*’*m in the menopause, so it*’*s logical.*’ *No, there was a huge drama before I got it sorted out.*”*(13112020)*

Even though participants had reached the end of their rope, they experienced this stage of life positively as a time when they learned how to set and protect their boundaries:

“*I think the older you get - and maybe it*’*s got something to do with the menopause* –*that certain things, I mean there comes a point when you have to clearly indicate your boundaries. You think* ‘*right, up to here and no further.*’ *I think it has something to do with having a short fuse and then you think* ‘*I don*’*t need to do it, so I won*’*t do it, I just won*’*t do it.*” (*Mariam, 50, Director small municipal organization for migrant women, 36 hours a week, 17112020)*

“*At the point when you become aware how much you can cope with, then you need to organize things differently, and I think a good way is when you are in the menopause you should you realize that this is the way it has to be, and you should not exceed these boundaries.*” *(Carola, 50, native Dutch, teacher, 32 hours a week, 13112020)*

Besides obvious physiological challenges participants described, we identified challenges both in paid work and private life: (1) *work environment and working conditions, and (2) burdens in private life*. Experiencing stressors from different sides simultaneously in a context that overwhelms them, and is not adapted to their needs, leads to them feeling exhausted.

### 3.1 “My fear is they will make fun of you” –Work environment and working conditions

The kind of work environment appears to matter to the women’s wellbeing. Various participants felt the need to conceal their menopause-related phenomena. Particularly in a work environment with younger and male colleagues, the visibility of these phenomena, such as hot flashes, was experienced as problematic:

“*Yes, it*’*s like they think you*’*re an attention seeker or something. Indeed, the sweating and the hot flashes that are bothering me more and more, it*’*s like you are ashamed of them or something, but it is actually purely the reaction of your colleagues, they just look but don*’*t discuss it, it doesn*’*t interest them either because they are 15 years younger than me.*” *(Sanne, 46, native Dutch woman, administrative assistant, 28 hours a week, 13112020)*

“*Then it*’*s like, at least that is my fear, like they will make fun of you. They*’*ll say* ‘*You know, the woman is in the menopause and will be no use for the rest* . . .  *the rest of the working day.*’ *And I really don*’*t want that to happen.*” *(Carola,13112020)*

In these examples we identified shame and the fear of not being taken seriously. At the same FGD, another participant who often works with younger colleagues had a different experience. Whereas others tried to conceal their phenomena, Annemarie (52), a native Dutch woman who works as a customer care support employee for 28 hours a week, was open about her menopausal status in order to be accepted:

“*I am very open myself and [* . . . *] but for example in our company*’*s culture, I see myself as a sort of a dinosaur among the youngsters and I just announce that the menopause is a fact, and then they just accept it as normal.*” (*13112020*)

In addition to work environment, various participants were in paid work in circumstances that were not attuned to their physiological challenges. When experiencing physiological challenges, such as sleeplessness, having a short fuse, and hot flashes, their working conditions, including inflexible working hours, high emotional work demands, and physically demanding work, affected participants wellbeing. Samantha (49) is a Surinamese woman and works as a patient food service assistant for 30 hours a week. Her work schedule, where late and early shifts alternate, is inflexible which often made her feel exhausted Reflecting on what these working conditions do to her wellbeing, Samantha said:

“*Y es, I am friendly with everyone you know, but I do notice, how do I put this nicely, that I quickly get angry, really become irritated* . . .  *at little things, I get irritated, little things.*” *(25112021)*

Another example is Sanne (46) who is a native Dutch woman who worked at an IT service assistance desk where she was switching between people and their computer problems all day. She thought this type of work has taken its toll:

“*That was very difficult for me, especially switching gear in the long-term. You do really want to help people, but it became like nagging or something, and it made me very irritable. I always stayed very friendly, but at home I became very angry and eventually burned out.*” *(13112020)*

In contrast to working conditions that seemed to negatively impact women’s wellbeing, participants mentioned work autonomy as a way to better manage their physiological challenges. Gülay (47) a Turkish woman who works as a university lecturer for 36 hours experiences her “academic freedom” as a great advantage in managing physiological challenges. She can organize her working hours herself, as long as she gets her work done. Gülay believed that autonomy can also offer a solution for employees in other jobs and sectors. However, if the workload remains the same, autonomy alone does not feel like a solution for everyone. Helen, for instance, said:

“*I can decide on my own working hours. I can start when I want and finish when I want. It is sometimes better for the client if I go in the evening. Actually I don*’*t think being able to choose my times makes that much difference, because the workload stays the same and sometimes I work longer because I can choose my own hours.*” *(25112021)*

Various participants were temporarily granted a high degree of autonomy by the employer, such as flexible working hours, in order to be able to anticipate on a challenging period, for instance when caring for a sick family member:

“*I was given the opportunity to act in a very free manner. [* . . . *] I have never not worked in a week, but sometimes say four hours or just a few hours in a day and I was free to leave when I wanted. And, yes, I see this as an enormous advantage.*” (Annemarie, 52, native Dutch, customer care support employee, 28 hours a week, *13112020*)

How participants value their work seemed also to play a role in wellbeing. Various participants perceived their work as an important source of energy, including Annemarie:

“*It was like an anchor for me. So it was really very important to continue to work, but I have talked about that. I was one of the few [* . . . *] who regarded work as keeping myself grounded, especially in this situation. Lots of people think the opposite, that it is best to stop (13112020*)

### 3.2 “I counted down to the weekend so I could rest, but I didn’t get any rest” –Burdens in private life

In addition to challenges in the workplace, during midlife most participants also had to deal with an increased burden in their private life, mainly from informal caring responsibilities. Helen felt like she was “burned out.” She had a hard time combining her informal care responsibilities for her mother with her job demands, as her emotional work demands are high:

“*My mother has Alzheimer*’*s and I look after her as well as continuing to work for 32 hours. I work in youth care, and I sometimes have very severe cases to deal with. If I don*’*t sleep very well I still have to go to work. You can*’*t only put 80% effort in with a family, or begin to handle a crisis or counsel a family [* . . . *]. On the other hand, caring for my mother [* . . . *] is very demanding, so, yes [* . . . *] I do find it difficult to do both. My mother was admitted for a month this September, but then I called in 50% sick [* . . . *] because all of a sudden I felt burned out. But only when she had been admitted. Before that I think I was just surviving.*” *(25112021)*

We also identified the challenging combination of informal care and high emotional work demands among participants in other FGDs. Nadia had to deal with a sick father and sister who died very close together. During this period her daughter was also ill, and she could no longer cope with the combination of continuing to work and these challenges in her private life: “*I counted down to the weekend so I could rest, but I didn*’*t get any rest*.” All participants who gave informal care indicated that this burden is mainly put on women. They experienced that caring responsibilities were not equally distributed among children or daughters within their families. For Moroccan participants who were also oldest daughter in their families, these expectations were self-evident:

“*I am the eldest, and a lot of things are expected of you, especially in our culture. As a Moroccan and the eldest, you have to do everything for your family.*” *(Zara, 49, Moroccan, cook in a small municipal organization for migrant women, 36 hours a week, 17112020)*

Only in the Ghanaian group were caring responsibilities relevant in a different manner. As their parents and parents-in-law still lived in the country of origin, these women had to bear financial caring responsibilities.

### 3.3 Measures to manage and try to reduce exhaustion

Besides taking various measures to suppress physiological challenges at work, such as using pain medication, participants took various measures to manage and try to reduce exhaustion, including finding a new job or negotiating different job tasks, and reducing working hours. When it became too much due to exhaustion, they started looking for a new job or wishes to find another job, or negotiated different job tasks:

“*Due to sleeping badly I can*’*t work all day. That*’*s why I have switched to homecare work, so I can work a few hours and then be at home again. I was actually a children*’*s day care worker for 20 years but that is all day. I couldn*’*t do it anymore. That*’*s why I changed my job.*” *(Betil, 54, Turkish, homecare worker, 16 hours a week, 18122020)*

“*I switched back to the classroom from internal management. I thought: nice and straightforward, a specific number of children. I will do my thing there and the rest can get on with it.*” *(Marie, 13112020*)

“*I want to have a job where I only work eight hours and then go home. I don*’*t want to continue taking responsibility.*” *(Zara, 17112020)*

This latter quote is from Zara, a cook in a small municipal organization for migrant women. Although cooking was her only job, she was assigned all kinds of other highly emotionally loaded tasks such as supporting asylum seekers in daily life activities. She no longer wanted this and was looking for a job with one clearly defined task. What kept her from applying was the fear of not being hired anywhere: “Companies won’t accept me with all my complaints.” The Ghanaian participants also experienced difficulties; they said that as a consequence of being a migrant without qualifications they had no options on the labor market other than a cleaning job:

“*But here, because of the language, we can’t go to school to have a good job. So our husband have more, let’s say they have, they work more than us, because for us, the only the cleaning and the other things are easy for us, because of the language. And here, if you don’t go to school here to have a diploma, and those kind of things, you can’t do a good job. Only the cleaning, cleaning, cleaning, cleaning, cleaning.*” *(Cheyenne, 50, Ghanaian, cleaner, 35 hours a week, 12122020)*

Nursel (43), a Turkish woman who works as a social worker for 28 hours a week, also gave up her political career due to experiencing multiple complaints and the fact that she is a single mother caring for her two children living at home:

“*I have been politically active since 2013, so after work was always going to meetings, reading and preparing for meetings, and at the beginning of last year I became a councillor, so as well as my 28-hour working week, I was doing other things that I enjoyed; they challenged me, you see, and I have had to give them up because I couldn*’*t do it anymore.*” *(18122020)*

Various participants indicated they had started reducing their working hours when they reached the point of not being able to continue working as they had done before. Nadia started to work four hours less each week, and is now getting more rest. She was able take this measure, because she is not the breadwinner of the household: “*The advantage is that I am not the breadwinner*. [. . . *] I am in the luxury position, and it is really a luxury, because I didn*’*t need to, I was able to because I am not the breadwinner*.” Another factor that enabled women to work less was not having children or grandchildren or other family members for whom they carried financial responsibility. Several participants, mainly with a Surinamese background, would like to reduce their working hours, but they considered their financial independence to be more important, mainly for maintaining a full pension.

## 4 Discussion

Our findings indicate that women in midlife experience exhaustion related to paid work and private life. Exhaustion occurs after a certain limit has been reached, both physically and mentally, when women have reached the end of their rope. Besides physiological challenges, we identified challenges both in paid work and private life: (1) *work environment and working conditions*, and (2) *burdens in private life.* Experiencing overwhelming stressors in differing domains simultaneously in a context that does not accommodate their needs, leads to feeling exhausted. Women take various measures to manage, and try to reduce exhaustion, including finding a new job or negotiating different job tasks, and reducing work hours.

The literature defines emotional exhaustion as a state comprising fatigue and loss of vitality [36], that can be understood as core component of burn-out [37]. While our study indicates that it is plausible that midlife women experience exhaustion, to our knowledge, there are only a few studies on exhaustion among women in this life phase. Lindeberg et al. (2011) found in a Swedish population that exhaustion was twice as common in women than in men [36], and psychosocial working conditions could only explain this difference to a limited extent. Verdonk et al. (2010) show that in the Netherlands highly-educated midlife women reported greater fatigue than other groups, reflected in sleeping problems, emotional exhaustion, doctor visits due to fatigue, and the need for recovery after work [5]. Converso et al. (2019) show that menopausal symptoms are strongly associated with exhaustion [37].

Experiences of exhaustion appear to be associated with underlying structural gendered processes. Firstly, both their bodily reproductive and post-reproductive stages are fully incorporated into women’s working lives. These female-specific life stages come with certain health challenges likely to be aggravated by an adverse work environment, i.e. working conditions insufficiently accommodating female-specific needs. Our findings show that work autonomy indicates whether women experience their menopause-related phenomena as problematic or not, and support Rees et al. (2021) in that the menopausal experience is shaped by menopausal symptoms and context, as well as by work environment [38]. Secondly, women more often work in under-resourced professions where chronic staff shortages are common [39]. This results in a high, often emotionally-demanded, workload. Thirdly, whether or not women do paid work, they continue to bear the brunt of unpaid care work [27]. Our findings indicate that informal caring responsibilities are highly gendered. Hence, we argue the possibility that exhaustion could have been prevented if women’s lives had been arranged differently. Unlike the growing number of studies providing evidence that the menopause negatively impacts work-related outcomes, we argue that –with the exception of women with severe health problems–as a physiological experience in itself menopause seems to be an insufficient explanation for women’s fatigue at this life stage.

Another finding is that midlife women seem to take various individual measures to deal with exhaustion which have consequences for their participation in paid work, and societal participation more broadly. This study showed that during midlife women’s participation in paid work rarely increased; it either remained the same, decreased, or they chose other jobs or job tasks, often below their competence levels. Our findings indicate that providing women in midlife with sufficient autonomy and responsibilities tailored to their individual circumstances may be important in preventing unwanted attrition, e.g., migration to lower-paid jobs, reducing work hours or prolonged absence through sick leave, which in turn impact women’s economic situation. Providing sufficient work autonomy, such as flexible working hours, as helpful way of maintaining participation is consistent with studies on work participation among workers with chronic conditions [40, 41].

A limitation is that two FGDs were largely natural groups whereas three were constructed groups. Leask et al. (2001) found differences in group dynamics, depth of interaction, and diversity between types of groups [42]. we also found that natural groups seemed to display a higher level of conformity, in particular in the Ghanaian group. However, this may also be due to the fact that all Ghanaian participants had the same profession and experienced similar barriers on the labor market in relation to their migration background. Another limitation is that –with the exception of one participant –we excluded women who are currently no longer participating in paid work. Therefore, we gained little insight into whether women experiencing exhaustion might have led to them abandoning paid work.

A strength is that we included a diverse group of women in order to explore a wide variety of experiences. Another strength was our methodological approach. We started each FGD with a case story based on participants own experiences, and by asking only one open question. In this way, the discussions remained very close to the experiences of the women.

Women deal with the situation by taking individual measures. However, we advocate the implementation of structural measures at the organizational level, preferably preventive in nature, in order to create more inclusive work environments for female workers at this life stage. We plea for the timely and proactive identification of exhaustion. Firstly, we recommend life-stage-oriented workers’ health surveillance (WHS) with specific attention to gender. One WHS aim is to monitor and promote the health of individual and groups of employees in relation to work [43]. WHS is intended to track both individual data and aggregated group data. Hence, as well as providing individual advice, WHS can lead to group-level feedback to the organization, enabling the employer and the Occupational Health Service to determine what gender-specific interventions are needed for workers in midlife.

Secondly, since our findings indicate that work autonomy is very important in preventing women from becoming exhausted, we recommend creating more work autonomy for women during midlife. Here, we make a distinction between structural autonomy and temporal work autonomy, for which an precondition seems to be an understanding attitude of the employer, and in particular, instrumental support by the direct supervisor. As confirmed in other studies on menopause and work [29, 38], line management should be well informed about female-specific challenges at this stage of life. To ensure that female workers are granted sufficient autonomy, it is important that future occupational health guidelines on menopause and work address this matter.

We recommend that further research includes a qualitative analysis of the structural position of women in order to determine those women in midlife who are most at risk of experiencing exhaustion due to background characteristics such as migratory background. Moreover, we suggest an intersectional quantitative analysis on a large cohort data set in order to examine the size of the group of women who reduce their participation in paid work or fully withdraw from the labor market. Intersectionality, originating in feminist scholarship [44], refers to how different aspects of identity, such as gender, ethnicity and social class, interact and influence people’s individual daily experiences.

## 5 Conclusion

This study indicates that the extent to which women experience exhaustion in midlife is associated with challenges in both paid work and private life. The underlying processes do not seem to reflect ‘just’ women’s individual problems, but are associated with a complex set of factors at structural level. Women who have reached their limit in this complex stage of life, where physiological-, work-related- and private life challenges continue to drain their energy, take several individual measures, including reducing their participation in paid work. However, we should ask ourselves how adequate these solutions really are. It may feel like a short-term solution, but for both the individual woman and society as a whole it is not a sustainable one.

## Ethics statement

The research proposal was submitted to and approved by the Medical Ethical Committee of Amsterdam UMC, location AMC, who decreed that a comprehensive evaluation was not required since this study was not subject to the Medical Research Involving Human Subjects Act (W20_381 # 20.426).

## Informed consent

Written informed consent was required from all individual participants included in the study prior to participation.

## Reporting guidelines

The consolidated criteria for reporting qualitative research was used (COREQ).

## Footnotes

^1^
We acknowledge that there is a debate about definitions and measures regarding sex and gender. Not all women menstruate or go through menopause, and not all people who menstruate are women. In this study, we use “women” to refer to those people who identify as women and who go through menopause.

## References

[1] Smits W , De Vries R , Veranderende beroepsloopbanen van mannen en vrouwen. Den Haag: CBS; 2013.

[2] CBS. Arbeidsdeelname en werkloosheid 2021 [Available from: http://statline.cbs.nl/Statweb/publication/?DM=SLNL&PA=0ned&D1=3-7,10-14&D2=l&D3=l&D4=166-181&HDR=T&STB=G1,G2,G3&VW=T

[3] Jones MK , Latreille PL , Sloane PJ , Staneva AV . Work-related health risks in Europe: Are older workers more vulnerable? Social Science & Medicine, 2013;88:18–29. 23702206 10.1016/j.socscimed.2013.03.027

[4] Strauss JR . Contextual infuences on women’s health concerns and attitudes toward menopause. Health & Social Work. 2011;36(2):121–7. 21661301 10.1093/hsw/36.2.121

[5] Verdonk P , Hooftman WE , van Veldhoven MJ , Boelens LR , Koppes LL . Work-related fatigue: the specific case of highly educated women in the Netherlands. International Archives of Occupational and Environmental Health. 2010;83(3):309–21. 19888593 10.1007/s00420-009-0481-y PMC2820214

[6] Bekker MH , Rutte CG , Van Rijswijk K . Sickness absence: a gender-focused review. Psychology, Health & Medicine. 2009;14(4):405–18. 10.1080/13548500903012830 19697251

[7] Van der Molen H , Kuijer P , De Groene G , Sorgdrager B , Lenderink A , Maas J , Brand T , Tamminga S . Kerncijfers beroepsziekten 2021. Amsterdam: NCVB; 2021.

[8] Verdonk P . Gender en arbeid. Werkstress, overspannenheid en burnout. In: Van Amelsvoort T, Bekker M, Van Mens-Verhulst J, Olff M, editor. Handboek Psychopathologie bij Vrouwen en Mannen Amsterdam (The Netherlands): Boom uitgeverij; 2019. p. 379-90.

[9] Griffiths A , MacLennan SJ , Hassard J . Menopause and work: an electronic survey of employees’ attitudes in the UK. Maturitas. 2013;76(2):155–9. 23973049 10.1016/j.maturitas.2013.07.005

[10] Sarrel P , Rousseau M , Mazure C , Glazer W . Ovarian steroids and the capacity to function at home and in the workplace. Annals of the New York Academy of Sciences. 1990;592:156–61. 2375580 10.1111/j.1749-6632.1990.tb30323.x

[11] Geukes M , Van Aalst M , Nauta M , Oosterhof H . The impact of menopausal symptoms on work ability. Menopause (New York, NY). 2012;19(3):278–82. 10.1097/gme.0b013e31822ddc97 21997498

[12] Raczkiewicz D , Bojar I , Humeniuk E . Work ability, functional exercise capacity and prevalence of obesity in perimenopausal and postmenopausal women with non-manual employment. International Journal of Occupational Safety and Ergonomics. 2019:1–9. 10.1080/10803548.2019.1676565 31584355

[13] Olajubu A , Olowokere A , Amujo D , Olajubu T . Influence of menopausal symptoms on perceived work ability among women in a Nigerian University. Climacteric: The Journal of the International Menopause Society. 2017;20(6):558–63. 28933968 10.1080/13697137.2017.1373336

[14] Hammam RA , Abbas RA , Hunter MS . Menopause and work–the experience of middle-aged female teaching staff in an Egyptian governmental faculty of medicine. Maturitas. 2012;71(3):294–300. 22226630 10.1016/j.maturitas.2011.12.012

[15] Kleinman N , Rohrbacker N , Bushmakin A , Whiteley J , Lynch W , Shah S . Direct and indirect costs of women diagnosed with menopause symptoms. Journal of Occupational and Environmental Medicine. 2013;55(4):465–70. 23532198 10.1097/JOM.0b013e3182820515

[16] Whiteley J , DiBonaventura M , Wagner JS , Alvir J , Shah S . The impact of menopausal symptoms on quality of life, productivity, and economic outcomes. Journal of Women’s Health. 2013;22(11):983–90. 10.1089/jwh.2012.3719 PMC3820128 24083674

[17] Geukes M , van Aalst M , Robroek S , Laven J , Oosterhof H . The impact of menopause on work ability in women with severe menopausal symptoms. Maturitas. 2016;90:3–8. 27282787 10.1016/j.maturitas.2016.05.001

[18] Whiteley J , Wagner J-S , Bushmakin A , Kopenhafer L , DiBonaventura M , Racketa J . Impact of the severity of vasomotor symptoms on health status, resource use, and productivity. Menopause (New York, NY). 2013;20(5):518–24. 10.1097/GME.0b013e31827d38a5 23403500

[19] DiBonaventura M , Wagner J , Alvir J , Whiteley J . Depression, quality of life, work productivity, resource use, and costs among women experiencing menopause and hot flashes: a cross-sectional study. The Primary Care Companion for CNS Disorders. 2012;14(6). 10.4088/PCC.12m01410 PMC3622540 23586001

[20] Bolge SC , Balkrishnan R , Kannan H , Seal B , Drake CL . Burden associated with chronic sleep maintenance insomnia characterized by nighttime awakenings among women with menopausal symptoms. Menopause (New York, NY). 2010;17(1):80–6. 10.1097/gme.0b013e3181b4c286 19730276

[21] Hardy C , Thorne E , Griffiths A , Hunter M . Work outcomes in midlife women: the impact of menopause, work stress and working environment. Women’s Midlife Health. 2018;4(1):3. 30766713 10.1186/s40695-018-0036-z PMC6298001

[22] Woods N , Mitchell E . Symptom interference with work and relationships during the menopausal transition and early postmenopause: observations from the Seattle Midlife Women’s Health Study. Menopause (New York, NY). 2011;18(6):654–61. 10.1097/gme.0b013e318205bd76 PMC9668245 21317821

[23] Jack G , Riach K , Bariola E . Temporality and gendered agency: Menopausal subjectivities in women’s work. Human Relations. 2019;72(1):122–43.

[24] Hardy C , Griffiths A , Thorne E , Hunter M . Tackling the taboo: talking menopause-related problems at work. 2019;12(1):28–38.

[25] Grandey A , Gabriel A , King E . Tackling taboo topics: a review of the three M s in working women’s lives. Journal of Management. 2020;46(1):7–35.

[26] Dillaway H . Living in Uncertain Times: Experiences of Menopause and Reproductive Aging. The Palgrave Handbook of Critical Menstruation Studies. 2020:253–68. 33347184

[27] Atria. De nieuwe mantelzorger v/m. Amsterdam: Atria; 2015.

[28] Broese-Van Groenou M , Schakel S , Tolkacheva N . Werk en mantelzorg: Een risico voor de psychische gezondheid? Tijdschrift voor arbeidsvraagstukken. 2015;31(4):393–410.

[29] Hardy C , Griffiths A , Hunter M . What do working menopausal women want? A qualitative investigation into women’s perspectives on employer and line manager support. Maturitas. 2017;101:37–41. 28539167 10.1016/j.maturitas.2017.04.011

[30] Krueger R . Focus groups: A practical guide for applied research. Thousand Oaks (United States): Sage publications; 2014. 280 p.

[31] Wong L . Focus group discussion: a tool for health and medical research. Singapore Med J. 2008;49(3):256–60. 18363011

[32] Tong A , Sainsbury P , Craig J . Consolidated criteria for reporting qualitative research (COREQ): a 32-item checklist for interviews and focus groups. International Journal for Quality in Health Care. 2007;19(6):349–57. 17872937 10.1093/intqhc/mzm042

[33] Strauss A , Corbin J . Basics of qualitative research techniques. Thousand Oaks (United States): Sage Publications, Inc.; 1998.

[34] Boeije H . Analyseren in kwalitatief onderzoek. Amsterdam (The Netherlands): Boom; 2005.

[35] [35[ Doyle S . Member checking with older women: A framework for negotiating meaning. Health Care for Women International. 2007;28(10):888–908. 17987459 10.1080/07399330701615325

[36] Lindeberg SI , Rosvall M , Choi B , Canivet C , Isacsson S-O , Karasek R , et al. Psychosocial working conditions and exhaustion in a working population sample of Swedish middle-aged men and women. European Journal of Public Health. 2011;21(2):190–6. 20504950 10.1093/eurpub/ckq039

[37] Converso D , Viotti S , Sottimano I , Loera B , Molinengo G , Guidetti G . The relationship between menopausal symptoms and burnout. A cross-sectional study among nurses. BMC Women’s Health. 2019;19(1):1–12. 31775724 10.1186/s12905-019-0847-6 PMC6882317

[38] Rees M , Angioli R , Coleman R , Glasspool R , Plotti F , Simoncini T , et al. European Menopause and Andropause Society (EMAS) and International Gynecologic Cancer Society (IGCS) position statement on managing the menopause after gynecological cancer: focus on menopausal symptoms and osteoporosis. International Journal of Gynecologic Cancer. 2020;30(4). 10.1136/ijgc-2020-001217 32046979

[39] UWV. Zorg. Factsheet Arbeidsmarkt: UWV; 2020 [Available from: https://www.uwv.nl/overuwv/Images/factsheet-arbeidsmarkt-zorg-maart2020.pdf

[40] Detaille S , Haafkens J , van Dijk F . What employees with rheumatoid arthritis, diabetes mellitus and hearing loss need to cope at work. Scandinavian Journal of Work, Environment & Health. 2003(2):134–42. 10.5271/sjweh.715 12718499

[41] Varekamp I , Van Dijk F . Workplace problems and solutions for employees with chronic diseases. Occupational Medicine. 2010;60(4):287–93. 20511269 10.1093/occmed/kqq078

[42] Leask J , Hawe P , Chapman S . Focus group composition: a comparison between natural and constructed groups. Australian and New Zealand Journal of Public Health. 2001;25(2):152–4. 11357912 10.1111/j.1753-6405.2001.tb01838.x

[43] Sluiter J , Weel A , Hulshof C . PMO: Leidraad voor preventief medisch onderzoek van werkenden. Utrecht: NVAB; 2013.

[44] Crenshaw K . Demarginalizing the intersection of race and sex: A black feminist critique of antidiscrimination doctrine, feminist theory and antiracist politics. University of Chicago Legal Forum. 1989:139–67.

